# The Comprehensive Effect of Depression, Anxiety, and Headache on Pain Intensity and Painkiller Use in Patients with Headache Analyzed by Unsupervised Clustering Using Machine Learning

**DOI:** 10.3390/biomedicines13061345

**Published:** 2025-05-30

**Authors:** Jong-Ho Kim, Minha Ahn, Jong-Hee Sohn, Sung-Mi Hwang, Jae-Jun Lee, Young-Suk Kwon

**Affiliations:** 1 Department of Anesthesiology and Pain Medicine, Chuncheon Sacred Heart Hospital, Hallym University College of Medicine, Chuncheon 24253, Republic of Korea; poik99@hallym.or.kr (J.-H.K.); h70sm@hallym.or.kr (S.-M.H.); 2 Institute of New Frontier Research, Hallym University College of Medicine, Chuncheon 24253, Republic of Korea; minha11@hallym.ac.kr (M.A.); deepfoci@hallym.or.kr (J.-H.S.); 3 Big Data Center, Chuncheon Sacred Heart Hospital, Hallym University College of Medicine, Chuncheon 24253, Republic of Korea; 4 Department of Neurology, Chuncheon Sacred Heart Hospital, Hallym University College of Medicine, Chuncheon 24253, Republic of Korea

**Keywords:** headache, artificial intelligence, clustering, depression, anxiety, quality of life, pain intensity, painkillers

## Abstract

**Background/Objectives**: Patients with headache experience depression, anxiety, and reduced quality of life, which are individually associated with pain intensity and painkiller use, but their comprehensive combined effect remains unclear. **Methods**: Comprehensive patient groups were formed based on unsupervised clustering using machine learning algorithms, and their associations were analyzed via ordinary least square regression. K-means and t-distributed stochastic neighbor embedding (t-SNE) combined with hierarchical density-based spatial clustering of applications with noise (HDBSCAN) were applied for clustering. **Results**: A total of 813 patients were subdivided via K-means clustering (2 clusters) and t-SNE + HDBSCAN clustering (4 clusters). In the K-means clustering, Cluster 1 showed significantly lower peak pain intensity (coefficient [95% CI]: −0.7 [−1 to −0.4]) and frequency of painkiller use (−2.3 [−3.4 to −1.3]) compared to Cluster 0. In the t-SNE + HDBSCAN clustering, Clusters 2 and 3 showed higher peak pain intensity (1.1 [0.5–1.7] and 1.6 [1.0–2.2], respectively) and more frequent painkiller use (2.5 [0.4–4.5] and 4.4 [2.2–6.7], respectively) than Cluster 1. **Conclusions**: The clustering approach successfully generated groups that reflected a comprehensive profile of depression-, anxiety-, and headache-related quality of life. The clusters demonstrated significant differences which can help better characterize patients based on their psychological and functional impact.

## 1. Introduction

In 2021, headaches affected approximately 3.1 billion people (40% of the global population), more commonly women. Headache is the third most common neurological disorder overall and across all age groups from 3 to 80 years old. Despite regional variations, headache remains a global public health issue that affects individuals across all races, income levels, and geographic regions [1,2].

Headaches are often associated with psychological conditions, potentially resulting in mental health problems such as depression and anxiety disorders [3,4,5], which have a global prevalence of 19.7% and 13.7%, respectively [6]. In particular, migraines are associated with an increased risk of depression, considerable disability, and reduced quality of life. Additionally, the frequency of headaches is also linked to greater disability and lower quality of life [7]. Notably, the relationship between migraine and anxiety is bidirectional; recurrent migraines can contribute to anxiety, while anxiety can exacerbate migraines [8].

Pain intensity and the frequency of medication use are critical clinical indicators in patients with headaches. Pain intensity is a key factor in assessing not only treatment efficacy, but also the impact of headaches on disability and quality of life [9]. It is also valuable for guiding therapeutic decisions and informing sample sizes for future studies [10]. Moreover, psychological factors, particularly depression and anxiety, have been linked to increased pain perception [11], with one study reporting a modest association between the three [12]. The frequency of headache medication use is equally important, especially because medication overuse can result in headache (i.e., rebound headaches), which may become chronic [13,14]. Anxiety and depression are associated with increased headache frequency, and frequent headaches can be a manifestation of underlying depression. Thus, the frequent use of over-the-counter analgesics has been linked to elevated levels of anxiety and depression [7,15,16,17].

The impact of headaches on the quality of life is a key aspect of a patient’s functional profile, reflecting the burden of headaches on individuals and society [18]. Accordingly, several studies suggest that greater pain from headaches is associated with a greater impact on the quality of life [19,20].

Although many studies have explored the associations of headache with depression, anxiety, and headache-related disability, these factors are typically analyzed independently. Clinically, however, these conditions often coexist in patients. Therefore, a comprehensive approach that simultaneously considers these variables would be more reflective of actual patient experiences.

Cluster analysis has recently become more common in headache research to identify distinct patient subgroups based on clinical and psychophysical characteristics. For instance, Di Antonio et al. performed a cluster analysis using psychophysical variables such as pressure pain thresholds and cervical range of motion, which involved identifying subgroups of migraine patients with differing levels of pain sensitivity and disability [21]. Similarly, Yalinay Dikmen et al. applied K-means clustering in a large clinical cohort of patients with cluster headache, revealing hidden phenotypic subtypes with different clinical features and treatment responses [22]. These studies highlight the utility of unsupervised machine learning methods in discovering clinically meaningful patterns among heterogeneous populations of patients with headaches.

Building on this foundation, the present study applied unsupervised clustering using machine learning algorithms to categorize patients with headache based on depression, anxiety, and functional impact, as measured using validated scales. Then, we analyzed the relationship of these clusters to two key clinical outcomes: peak pain intensity and the frequency of analgesic use. This study aimed to evaluate whether these psychological and functional domains could be used to stratify patients in terms of pain-related behaviors, which can be valuable in individualized management strategies.

## 2. Materials and Methods

### 2.1. Study Design, Ethical Approval, and Informed Consent

This retrospective study was conducted at Chuncheon Sacred Heart Hospital, College of Medicine, Hallym University, Chuncheon, Republic of Korea, and was approved by the Clinical Research Ethics Committee of the same institution (IRB No. 2025-03-021). The study utilized registered data from an ongoing research project (“Development of a Quantification and Management System for Pain Using Artificial Intelligence-Based Multimodal Registration Technology”), collected from 1 April 2023 to 13 February 2024. Since informed consent for data use was obtained from the participants in the main study, the need for additional consent in this retrospective analysis was waived by the ethics committee.

### 2.2. Data Source

The primary project is registered in the Korean Clinical Trial Database (http://cris.nih.go.kr/cris/index/index.do; KCT0008684; accessed on 21 March 2024). The primary project included adult patients aged 19–80 years with a chief complaint of headache consulting at the outpatient, inpatient, or emergency departments of Chuncheon Sacred Heart Hospital. Although the disease codes differed across patients, headache was the predominant presenting symptom in all cases. The inclusion and exclusion criteria are described in previous publications based on the same dataset [23,24].

### 2.3. Primary Outcomes

The primary outcomes were (1) peak headache pain intensity and (2) the frequency of painkiller use. Peak pain intensity was measured using a visual analog scale ranging from 0 to 10. The frequency of painkiller use was defined as the number of days wherein painkillers (i.e., any headache medication) were taken in the previous month, including nonsteroidal anti-inflammatory drugs, acetaminophen, opioids, triptans, or muscle relaxants prescribed for headache relief.

### 2.4. Clustering Factors and Other Headache-Associated Variables

Data regarding headache-associated variables were collected using surveys administered in the main study, and the survey instruments were described previously [23]. Three variables were used for clustering:

- Severity of headache impact on quality of life, based on the Headache Impact Test-6 (HIT-6; range: 36–78)
- Depression, based on the Patient Health Questionnaire-9 (PHQ-9; range: 0–27)
- Anxiety, based on the Generalized Anxiety Disorder 7-Item Scale (GAD-7; range: 0–21)

These three variables were standardized prior to clustering. No additional variables were used in the clustering process.

The following clinical variables were collected for descriptive and covariate analyses: age, sex, diagnosis of headache, duration of headache history (days), average headache attack duration, and average frequency of headache (per month). Headache was further categorized as either migraine, tension-type headache, or other causes; this was based on the classification of disease codes and clinical assessment. Probable diagnoses were also included in each category of headache. Further details regarding the diagnostic criteria and categorization were described in our previous study [24]. The average headache attack frequency was categorized as <1, 1–3, 4–11, and ≥12 times per month. The average headache attack duration was categorized as <30 min, 30 min to 4 h, 4–72 h, and ≥72 h.

### 2.5. Data Preprocessing

Patients with missing, outlier, or duplicate data (e.g., from follow-up visits) were excluded. HIT-6, PHQ-9, and GAD-7 scores were standardized prior to clustering.

### 2.6. Clustering

Clustering was performed using (1) K-means clustering and (2) t-distributed stochastic neighbor embedding combined with hierarchical density-based spatial clustering of applications with noise (t-SNE + HDBSCAN). For K-means clustering, the optimal number of clusters was determined using the Elbow method, Silhouette Scores, the Calinski–Harabasz Index, and the Davies–Bouldin Index. For t-SNE + HDBSCAN clustering, the optimal number of clusters was automatically estimated based on the data density and cluster stability. The input for t-SNE consisted of three standardized variables (HIT-6, PHQ-9, and GAD-7), with the resulting two-dimensional embedding used for clustering. The perplexity parameter was set to 50; this determines the number of nearest neighbors considered during t-SNE. The minimum cluster size for HDBSCAN was set to 35. Noise points (i.e., data not assigned to any cluster) were excluded from subsequent analyses. The implementations of K-means and t-SNE were based on the scikit-learn Python library (version 1.6.1) [25,26], while HDBSCAN was performed using the HDBSCAN Python package (version 0.8.40) [27,28].

### 2.7. Statistical Analysis

Continuous and categorical variables were summarized using the median (interquartile range) and n (%), respectively. The primary outcomes were compared between clusters via *t*-test or one-way analysis of variance (ANOVA), as appropriate, and visualized using boxplots.

To evaluate the association between cluster membership and the primary outcomes, ordinary least squares (OLS) regression was performed using each outcome as the dependent variable. The cluster was treated as a categorical independent variable using dummy coding, with Clusters 0 and 1 as the reference group in K-means clustering and t-SNE + HDBSCAN clustering, respectively. All models were adjusted for the following potential confounders: age, sex, headache diagnosis, duration of headache history (in days), average attack duration, and average attack frequency. In addition, the primary outcomes were included as covariates for each other to account for potential bidirectional influence (i.e., peak pain was included when analyzing painkiller frequency, and vice versa). Bonferroni correction was applied for multiple comparisons across the two primary outcomes, with statistical significance set at α = 0.025. All statistical analyses and visualization were performed using Python 3.11.11 on Google Colaboratory (Google LLC, Mountain View, CA, USA), a cloud-based computing environment.

### 2.8. Use of Generative Artificial Intelligence (AI)

Generative AI (ChatGPT, version: GPT-4o; OpenAI, San Francisco, CA, USA; accessed 11 April 2025) was used to assist with English language editing during manuscript preparation in the first submission.

## 3. Results

**Figure 1:**
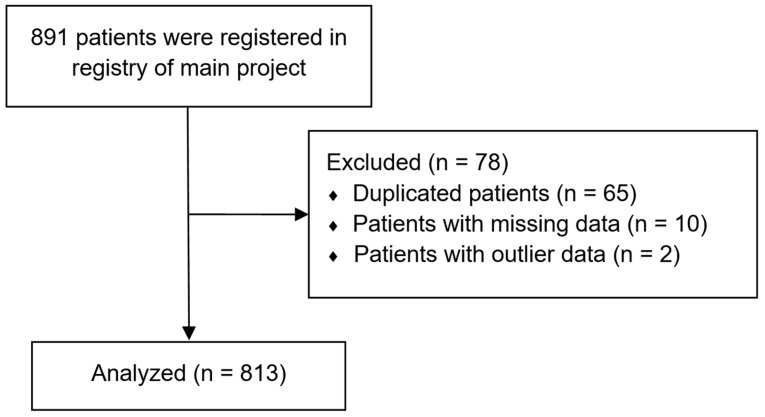
The patient selection flowchart.

A total of 891 patients were enrolled in the registry of the main project, and 78 patients with missing or duplicate data were excluded. The final analysis included 813 patients. The patient selection flowchart is summarized in Figure 1.

**Figure 2:**
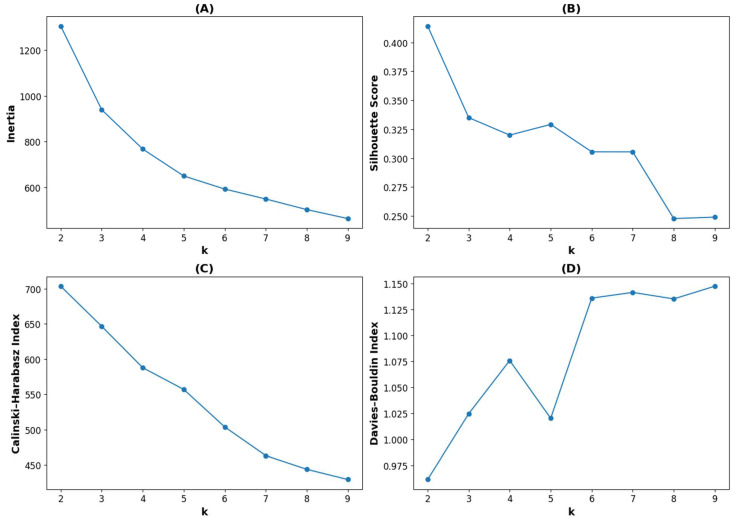
Dependence of internal validation metrics on the number of clusters (k) in K-means clustering. (**A**) Inertia (Elbow method); (**B**) Silhouette Score; (**C**) Calinski–Harabasz Index; (**D**) Davies–Bouldin Index. Better clustering is indicated by higher values in the Silhouette Score and Calinski–Harabasz Index, whereas lower values are preferable for the Davies–Bouldin Index. The Elbow method identifies an inflection point in intra-cluster inertia. These metrics were used collectively to determine the optimal number of clusters.

To determine the optimal number of clusters for K-means clustering, four internal validation metrics were evaluated: the Elbow method, Silhouette Score, Calinski–Harabasz Index, and Davies–Bouldin Index (Figure 2). Based on the Elbow method, intra-cluster inertia begins to plateau at either k = 5 or 6, representing an inflection point. Meanwhile, the Silhouette Score and Calinski–Harabasz Index both reached their maximum at k = 2, indicating that having 2 clusters achieved the best balance between intra-cluster cohesion and inter-cluster separation since both of these metrics favor higher values for better clustering. The Davies–Bouldin Index reached its minimum at k = 2; this favors lower values for better clustering quality by minimizing intra-cluster variance and maximizing inter-cluster separation. Collectively, these results suggest that k = 2 is the optimal number of clusters, considering both statistical validation and clinical interpretability, and this was, therefore, used for the final K-means clustering.

**Figure 3:**
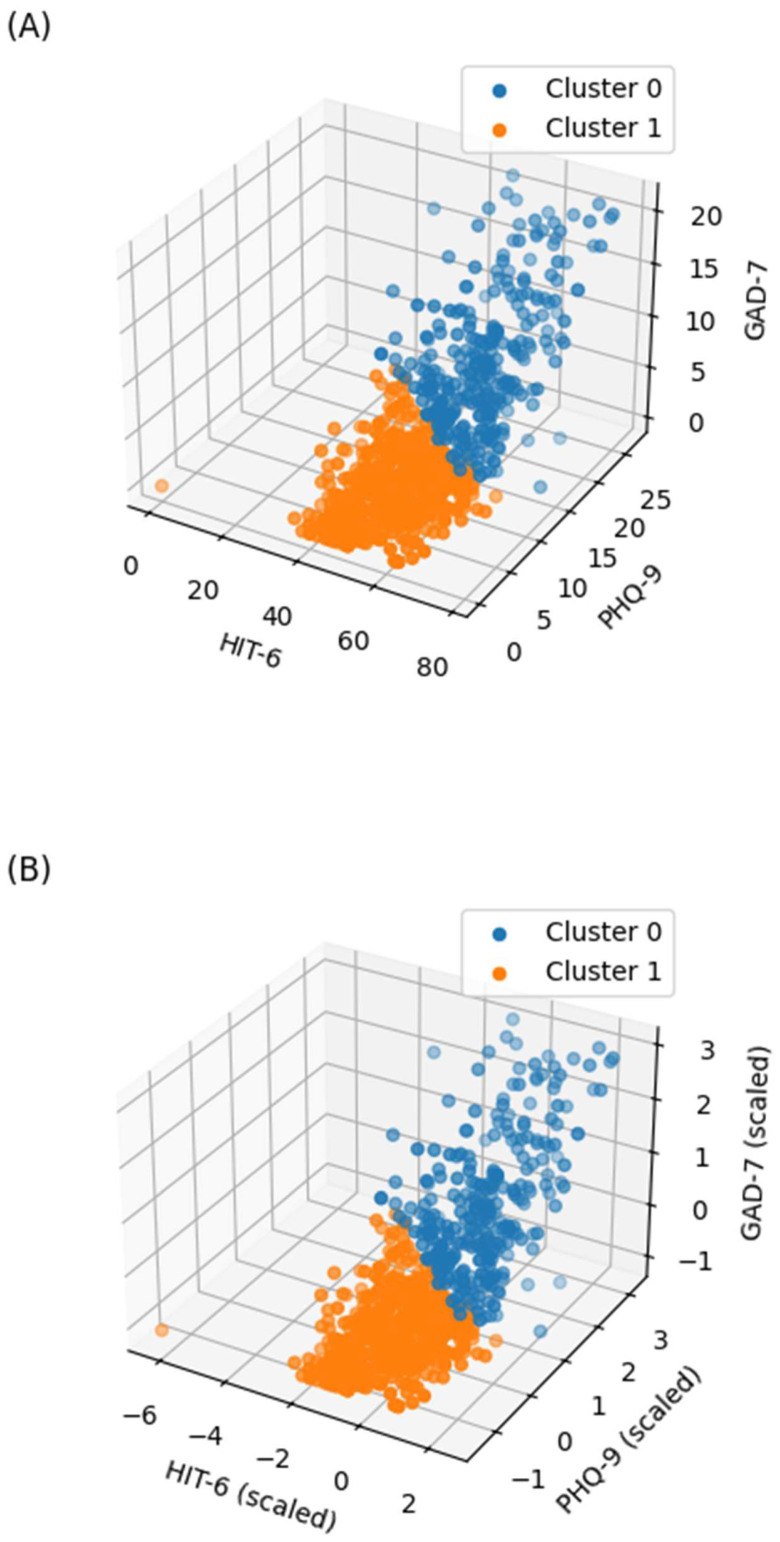
Distribution of HIT-6, PHQ-9, and GAD-7 scores based on K-means clustering. (**A**) Cluster distribution for raw data (based on scaled K-means); (**B**) cluster distribution for scaled data (same labels). HIT-6, Headache Impact Test-6; PHQ-9, Patient Health Questionnaire-9; GAD-7, Generalized Anxiety Disorder 7-Item Scale.

**Figure 4:**
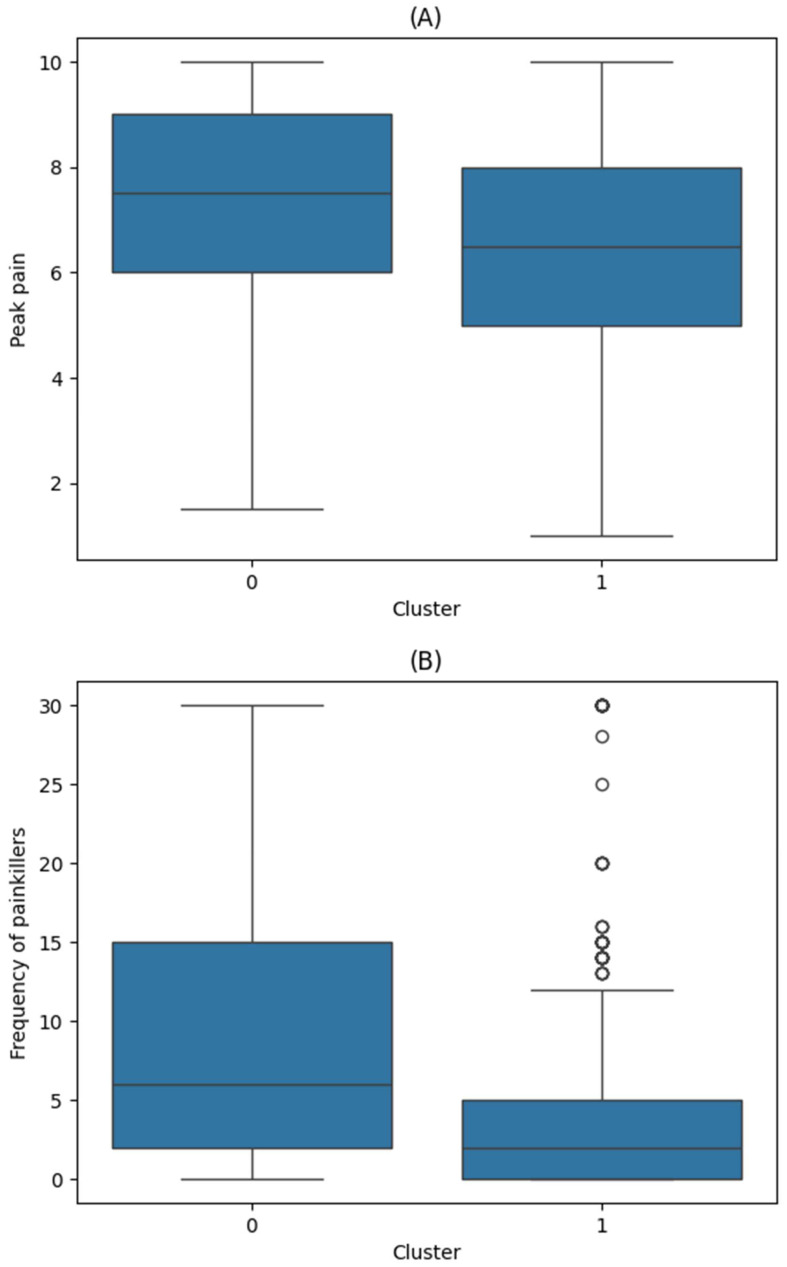
Distribution of primary outcomes by K-means cluster. (**A**) Peak pain intensity (mean; Cluster 0: 7.3; Cluster 1: 6.3; *p* < 0.001). (**B**) Frequency of painkiller use (mean; Cluster 0: 9.7; Cluster 1: 4.4; *p* < 0.001). Values are visualized using boxplots; *p*-values are from independent *t*-tests.

**Figure A1:**
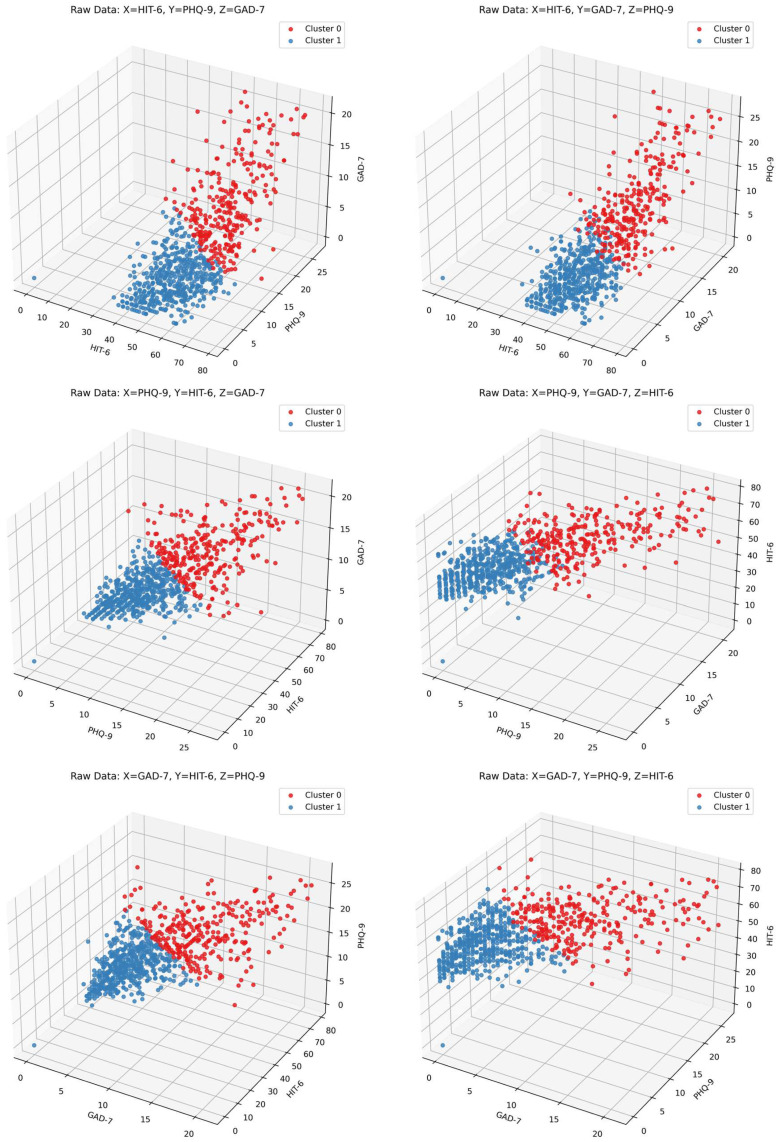
The distribution of HIT-6, PHQ-9, and GAD-7 scores based on K-means clustering with varying axis combinations. HIT-6, Headache Impact Test-6; PHQ-9, Patient Health Questionnaire-9; GAD-7, Generalized Anxiety Disorder 7-Item Scale.

The distribution of the HIT-6, PHQ-9, and GAD-7 scores by cluster is shown in Figure 3 and Figure A1 (Appendix A). The patient characteristics according to the K-means clusters are summarized in Table 1. Both peak pain intensity and frequency of painkiller use demonstrated significant differences between the clusters (*p* < 0.001 for both; Figure 4).

In the OLS regression analysis for K-means clusters, Cluster 1 had a significantly lower peak pain intensity (coefficient: −0.8, *p* < 0.001) and frequency of painkiller use (coefficient: −2.3, *p* < 0.001) compared to Cluster 0. The detailed results of the regression analysis are presented in Table 2.

**Figure 5:**
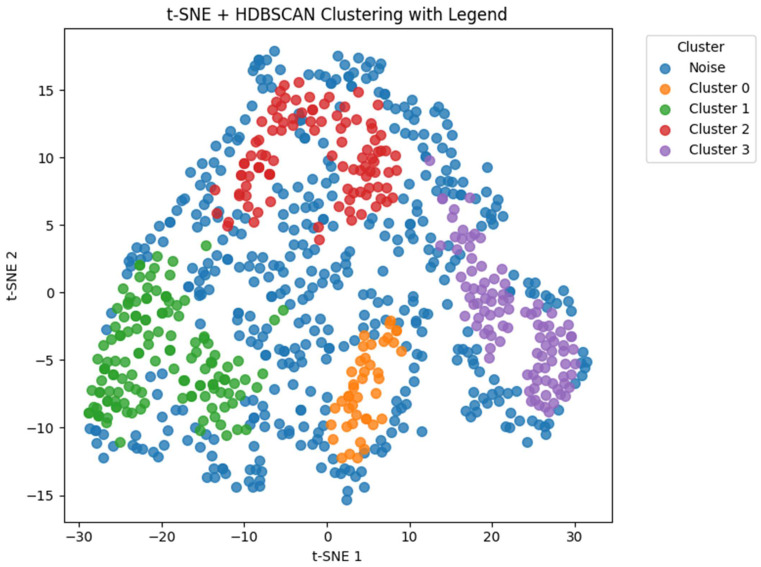
Distribution of clusters identified via HDBSCAN after dimensionality reduction using t-SNE. HDBSCAN, hierarchical density-based spatial clustering of applications with noise; t-SNE, t-distributed stochastic neighbor embedding.

The HDBSCAN clustering results after dimensionality reduction using t-SNE are visualized in Figure 5. A total of 4 clusters were identified, excluding 435 data points classified as noise. Patient characteristics of the remaining data according to the t-SNE + HDBSCAN clusters are shown in Table 3.

**Figure 6:**
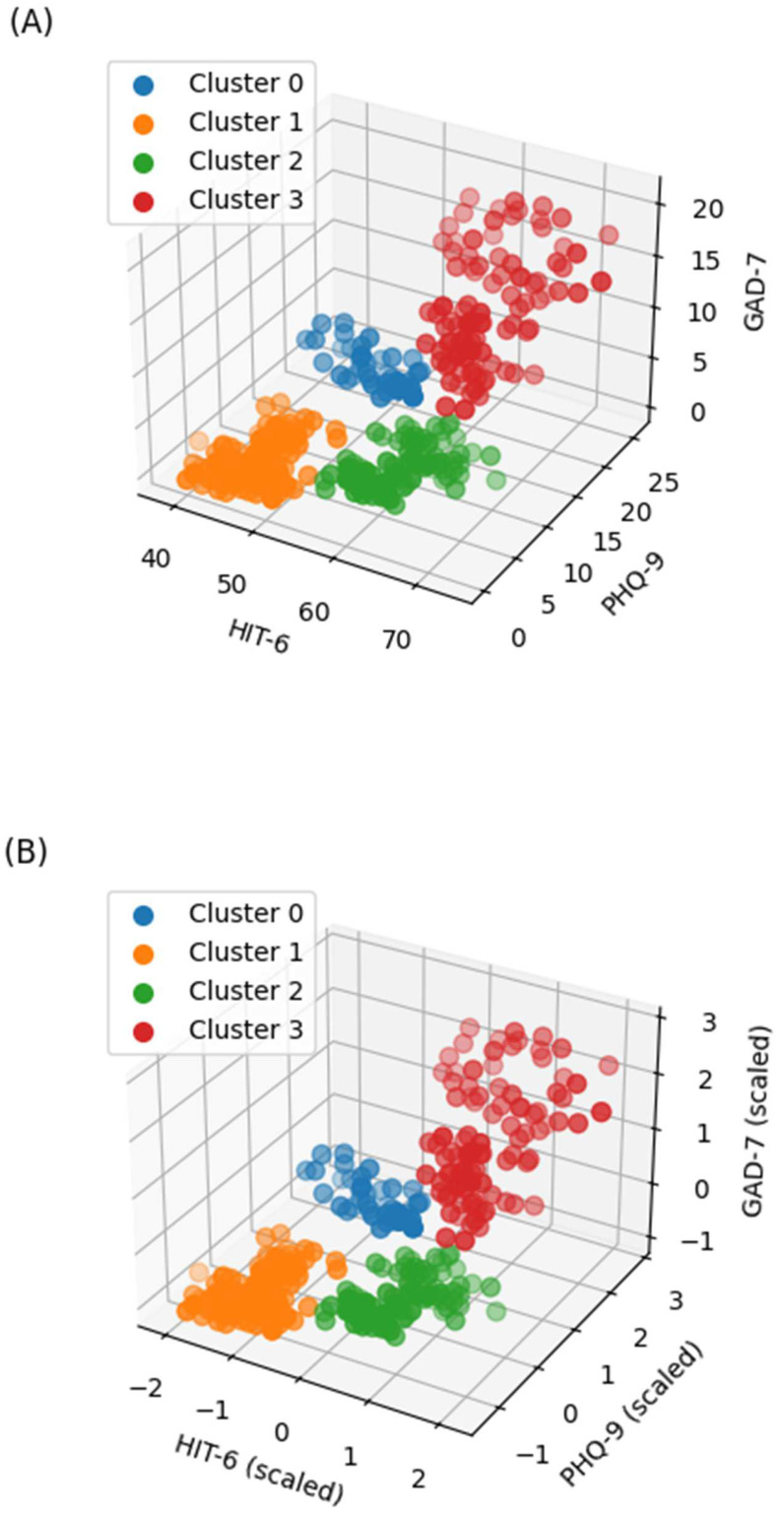
Distribution of HIT-6, PHQ-9, and GAD-7 scores based on t-SNE + HDBSCAN after noise removal. (**A**) Cluster distribution in raw data; (**B**) cluster distribution in scaled data. HIT-6, Headache Impact Test-6; PHQ-9, Patient Health Questionnaire-9; GAD-7, Generalized Anxiety Disorder 7-Item Scale; t-SNE, t-distributed stochastic neighbor embedding; HDBSCAN, hierarchical density-based spatial clustering of applications with noise.

**Figure 7:**
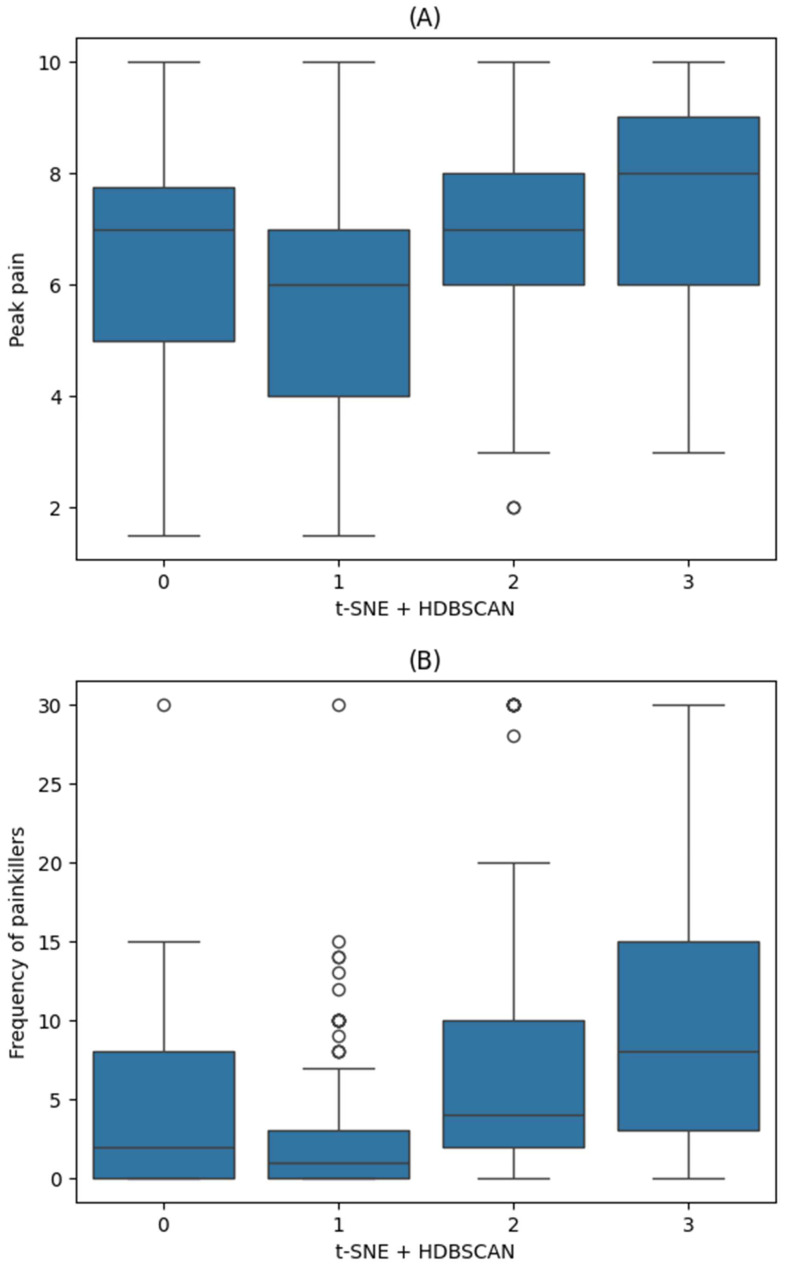
Distribution of primary outcomes by clusters identified through t-SNE + HDBSCAN. (**A**) Peak pain intensity (mean; Cluster 0: 6.4; Cluster 1: 5.9; Cluster 2: 7.0; Cluster 3: 7.5); (**B**) frequency of painkiller use (mean; Cluster 0: 4.4; Cluster 1: 2.6; Cluster 2: 7.6; Cluster 3: 10.6). Both outcomes demonstrated significant differences among clusters (*p* < 0.001, one-way ANOVA with Bonferroni correction). t-SNE, t-distributed stochastic neighbor embedding; HDBSCAN, hierarchical density-based spatial clustering of applications with noise; ANOVA, analysis of variance.

**Figure A2:**
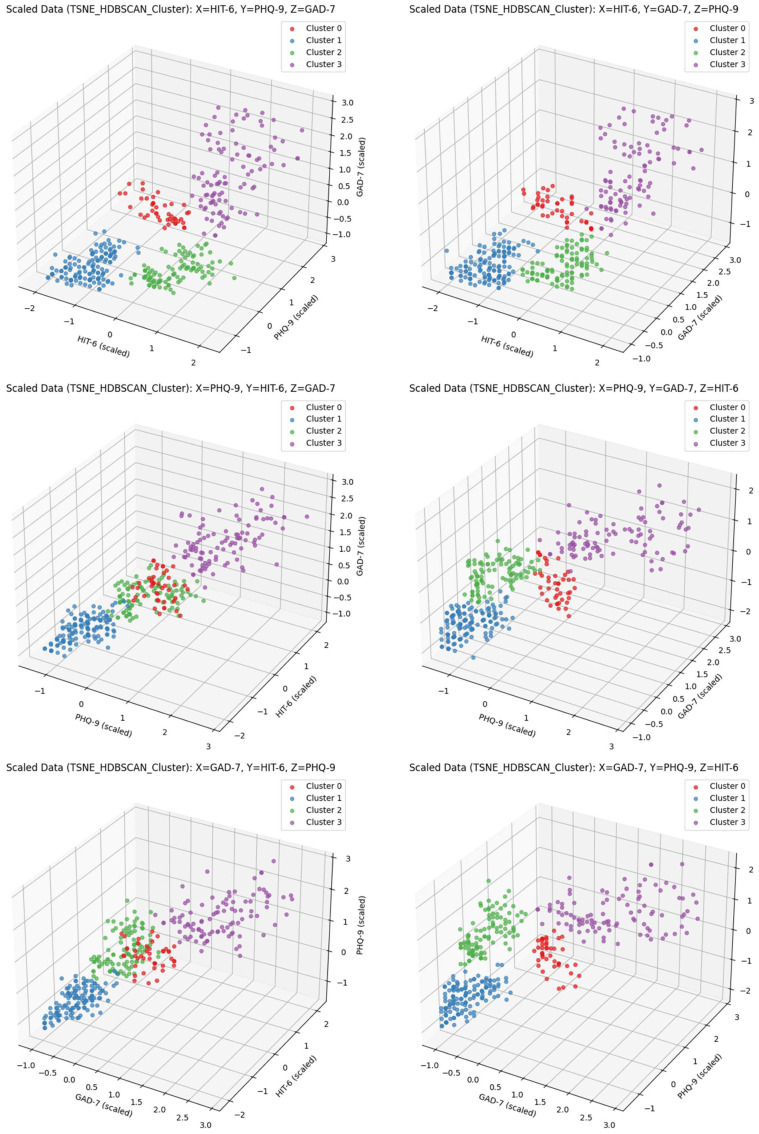
The distribution of HIT-6, PHQ-9, and GAD-7 scores based on t-SNE + HDBSCAN clustering with varying axis combinations. HIT-6, Headache Impact Test-6; PHQ-9, Patient Health Questionnaire-9; GAD-7, Generalized Anxiety Disorder 7-Item Scale; t-SNE, t-distributed stochastic neighbor embedding; HDBSCAN, hierarchical density-based spatial clustering of applications with noise.

The distributions of HIT-6, PHQ-9, and GAD-7 across the HDBSCAN clusters are illustrated in Figure 6 and Figure A2 (Appendix A). Both peak pain intensity and painkiller use demonstrated significant differences among the clusters (*p* < 0.001 for both; Figure 7).

**Table 3:** Unadjusted descriptive characteristics of patients by clusters identified via t-SNE + HDBSCAN after excluding noise.

		Cluster 0 (n = 43)	Cluster 1 (n = 136)	Cluster 2 (n = 101)	Cluster 3 (n = 98)
Age		51 (46, 61)	53 (40, 62)	43 (31, 58)	44 (29, 57)
Sex	Male	14 (32.6)	61 (44.9)	39 (38.6)	31 (31.6)
	Female	29 (67.4)	75 (55.1)	62 (61.4)	67 (68.4)
Diagnosis	Migraine	5 (11.6)	13 (9.6)	28 (27.7)	15 (15.3)
	TTHA	10 (23.3)	26 (19.1)	13 (12.9)	24 (24.5)
	Other headache	28 (65.1)	97 (71.3)	60 (59.4)	59 (60.2)
Duration of headache history (days)		365 (26, 1460)	60 (7, 1095)	730 (30, 3650)	730 (41, 3468)
Average frequency of headache attacks (times per month)	<1	0 (0)	9 (6.6)	0 (0)	0 (0)
	1–3	8 (18.6)	32 (23.5)	18 (17.8)	6 (6.1)
	4–11	23 (53.5)	70 (51.5)	35 (34.7)	31 (31.6)
	≥12	12 (27.9)	25 (18.4)	48 (47.5)	61 (62.2)
Average headache attack duration	<30 min	5 (11.6)	42 (30.9)	3 (3)	2 (2)
	30 min to 4 h	10 (23.3)	38 (27.9)	15 (14.9)	25 (25.5)
	4–72 h	16 (37.2)	29 (21.3)	44 (43.6)	31 (31.6)
	≥72 h	12 (27.9)	27 (19.9)	39 (38.6)	40 (40.8)

Values are presented without statistical comparisons since the table summarizes group-level characteristics only. Statistical comparisons of the primary outcomes are presented separately in Figure 7 and Table 4. TTHA, tension-type headache; t-SNE, t-distributed stochastic neighbor embedding; HDBSCAN, hierarchical density-based spatial clustering of applications with noise.

**Table 4:** Results of ordinary least squares regression based on t-SNE + HDBSCAN clustering.

		Peak Pain Intensity		Frequency of Painkiller Use	
		Coef (95% CI)	*p*	Coef (95% CI)	*p*
Clusters	Reference: Cluster 1				
	Cluster 0	0.6 (−0.1, 1.2)	0.098	0.8 (−1.6, 3.2)	0.52
	Cluster 2	1.1 (0.5, 1.7)	<0.001	2.5 (0.4, 4.5)	0.02
	Cluster 3	1.6 (1, 2.2)	<0.001	4.4 (2.2, 6.7)	<0.001
Age (years)		0 (0, 0)	0.116	0 (−0.1, 0)	0.317
Sex	Female	0.6 (0.2, 1)	0.007	−0.7 (−2.2, 0.8)	0.375
Diagnosis	Reference: other headache				
	Migraine	0.3 (−0.3, 0.8)	0.367	1.1 (−0.9, 3.2)	0.281
	TTHA	−0.2 (−0.7, 0.3)	0.419	0.2 (−1.7, 2)	0.844
Average frequency of headache attacks (times per month)	Reference: <1				
	1–3	−1.3 (−2.6, 0.1)	0.066	1.2 (−3.6, 6.1)	0.624
	4–11	−1.8 (−3.1, −0.5)	0.008	2.8 (−1.9, 7.5)	0.243
	≥12	−2 (−3.3, −0.6)	0.004	6.5 (1.7, 11.3)	0.008
Average headache attack duration	Reference: <30 min				
	30 min to 4 h	0 (−0.7, 0.7)	0.987	1.4 (−1, 3.9)	0.245
	4–72 h	−0.2 (−0.9, 0.5)	0.632	0.1 (−2.3, 2.6)	0.916
	≥72 h	−0.1 (−0.8, 0.6)	0.771	3.8 (1.4, 6.3)	0.002
Duration of headache history (days)		0 (0, 0)	0.465	0 (0, 0)	0.428
Frequency of painkillers/Peak pain intensity *		0 (0, 0.1)	0.017	0.4 (0.1, 0.8)	0.017

t-SNE, t-distributed stochastic neighbor embedding; HDBSCAN, hierarchical density-based spatial clustering of applications with noise; CI, confidence interval; coef, coefficient; TTHA, tension-type headache. * If the dependent variable is peak pain intensity, the coefficient and *p*-value refer to the effect of painkiller use, and vice versa.

In the OLS regression analysis for t-SNE + HDBSCAN clustering, Clusters 2 and 3, when compared to Cluster 1, both had significantly higher peak pain intensities (coefficients: 1.1 and 1.6, respectively; both *p* < 0.001) and more frequent painkiller (coefficients: 2.5 and 4.4, respectively; *p* = 0.02 and *p* < 0.001). Detailed regression results for HDBSCAN clustering are also shown in Table 2.

## 4. Discussion

In this study, 813 patients were clustered using unsupervised machine learning algorithms. When using K-means clustering, the patients were divided into two groups. Most patients in Cluster 1 scored lower on the depression (PHQ-9) and anxiety (GAD-7) scales versus those in Cluster 0. Additionally, Cluster 1 also had lower peak pain intensity and painkiller use than Cluster 0. When using t-SNE + HDBSCAN clustering, Cluster 0 was characterized by higher depression and anxiety scale scores than Cluster 1. In comparison to Cluster 1, Cluster 2 scored higher on the functional impact scale (HIT-6), while Cluster 3 had higher scores across all three indices (i.e., functional impact, depression, and anxiety). Both Cluster 2 and Cluster 3 were associated with increased peak pain intensity and more frequent use of painkillers.

In line with this, previous studies have shown that mood disorders such as depression and anxiety can exacerbate pain perception [11,29,30]. Among patients with chronic pain, the most common psychological comorbidity is depression, which can intensify if pain worsens [30,31], followed by anxiety [30]. Notably, the prevalence of anxiety disorders in populations experiencing pain is approximately twice that of the general population [32].

Pain intensity is closely linked to headache-related disability, which are both influenced by emotional states [19,33,34]. Pain-related disability can be worsened by an increased focus on pain or exaggeration of pain perception [35]. In our findings, clusters with more severe depression, anxiety, and functional impact tended to report higher peak pain intensity. However, the difference in pain intensity between the K-means clusters was <1 point on the pain scale. Since this finding likely has limited clinical significance, caution is warranted in interpreting these findings for clinical application.

Previous studies have linked the frequent use of painkillers with a higher risk of depression [36]. Although our study does not assess causality, the use of painkillers was significantly greater in Cluster 3, which is characterized by higher depression scores, compared to Cluster 1 (adjusted coefficient: 4.4 [95% CI: 2.2–6.7]). This pattern is in line with previous reports. Notably, the relationship between analgesic use (particularly opioids) and depression is bidirectional [37,38]. In other words, those with depression are more likely to misuse opioids, and long-term opioid use can also contribute to the development of depression. Concrete evidence has shown that 90–180 days of opioid use increases the risk of depression by 25%, while usage exceeding 180 days increases the risk by 50% [36]. Additionally, anxiety is also linked to the use of both opioids and analgesics in general. This relationship may be bidirectional as well, indicating that psychological states can influence medication use and vice versa [17,39,40]. Moreover, somatic symptoms of anxiety and depression (e.g., sleep disturbance, muscle tension) have been associated with increased analgesic use [17], particularly in women and in those with severe symptoms [15]. The fear of pain itself can also drive the frequent use of painkillers [41]. Factors such as socioeconomic status and physical activity influence the impact of headache and analgesic use [42,43,44,45], with physical inactivity linked to higher analgesic use in women [43]. Polypharmacy may lead to undesirable health outcomes such as increased morbidity and mortality, as well as reduced quality of life [46].

The relationships between pain intensity, analgesic use, depression, anxiety, and headache-related quality of life are complex and interdependent [11,19,29,30,31,32,33,34,35,47,48,49,50,51]. Because these factors influence each other, their collective impact is difficult to assess using conventional analytical methods. This complexity can hinder clinical decision-making as well. Clustering methods can address this issue by simplifying these multidimensional relationships [52]. We applied two clustering techniques commonly used in medical research: K-means and t-SNE + HDBSCAN [53,54,55,56,57].

K-means clustering is a widely used, iterative algorithm that groups data by minimizing the sum of the squared distances between data points and their cluster centers [58,59]. In our study, K-means clustering was able to summarize these three dimensions into two clusters using objective criteria. Despite the simplicity of this approach, cluster separation was clear, with statistically significant inter-group differences in outcomes. Furthermore, the results were clinically interpretable, suggesting that K-means clustering resulted in reliable insights.

However, t-SNE + HDBSCAN clustering provides a more refined approach. The first component, t-SNE, is a technique for visualizing high-dimensional data by embedding it in a low-dimensional space while preserving the structure of the data relationships. Probability distributions are used to represent similarities between data points [60,61]. Second, HDBSCAN, which is an extension of DBSCAN, improves clustering robustness by eliminating the need to manually set the epsilon parameter, enabling the detection of clusters with variable density [62,63]. However, HDBSCAN can classify data points as noise if they do not belong to any high-density region [64]. In our study, t-SNE + HDBSCAN clustering yielded four clusters, which provides a more granular view than K-means clustering. These clusters showed distinct distributions in terms of functional impact, depression, and anxiety, also demonstrating significantly different clinical outcomes. These well-separated and detailed clusters can be useful in developing more personalized approaches to the management of headaches.

This study comprehensively grouped depression, anxiety, and headache-related quality of life via unsupervised clustering with machine learning algorithms, enabling a better analysis of pain-related outcomes among patients with headache. However, this study has several limitations. First, the data were obtained from a single center, thereby limiting the generalizability of these findings, which need further external validation with data from other institutions. Second, the study population included all types of headache disorders without conducting subgroup analyses according to diagnosis because of the insufficient sample sizes. Although our findings reflect the general associations between psychological factors and headache burden, these may not accurately represent patients with specific diagnoses (e.g., migraine or tension-type headache). This warrants further studies with clustering and outcome analysis by headache subtype in order to optimize individual treatment strategies. Third, during t-SNE + HDBSCAN clustering, 435 patients (53.5%) were classified as noise and thereby excluded. Since these data points were not characterized in subsequent analyses, their clinical relevance remains unclear. Future research should explore additional methods to analyze and interpret noise data.

## 5. Conclusions

Clustering approaches were used to comprehensively analyze anxiety, depression, and headache-related quality of life in patients with headaches. Each cluster showed distinct profiles with significant differences in peak pain intensity and painkiller use. This method of clustering can help in the development of individualized approaches to treating headaches. Nevertheless, further efforts are needed to externally validate this data and to better analyze noise in high-dimensional data.

## Figures and Tables

**Table 1:** Unadjusted patient characteristics based on K-means cluster membership.

		Cluster 0 (n = 291)	Cluster 1 (n = 522)
Age		47 (33.5, 58)	49 (36, 61)
Sex	Male	93 (32)	190 (36.4)
	Female	198 (68)	332 (63.6)
Diagnosis	Migraine	58 (19.9)	90 (17.2)
	TTHA	61 (21)	74 (14.2)
	Other headache	172 (59.1)	358 (68.6)
Duration of headache history (days)		1095 (60, 3650)	97.5 (10, 1825)
Average frequency of headache attacks (times per month)	<1	0 (0)	11 (2.1)
	1–3	37 (12.7)	137 (26.2)
	4–11	93 (32)	235 (45)
	≥12	161 (55.3)	139 (26.6)
Average headache attack duration	≤30 min	21 (7.2)	87 (16.7)
	30 min to 4 h	53 (18.2)	106 (20.3)
	4–72 h	100 (34.4)	181 (34.7)
	≥72 h	117 (40.2)	148 (28.4)

Values represent descriptive statistics only (mean [SD] or n [%]); no statistical comparisons were performed.

**Table 2:** Results of the ordinary least squares regression based on K-means clustering.

		Peak Pain Intensity		Frequency of Painkiller Use	
		Coef (95% CI)	*p*	Coef (95% CI)	*p*
Cluster	Reference: Cluster 0				
	Cluster 1	−0.7 (−1, −0.4)	<0.001	−2.3 (−3.4, −1.3)	<0.001
Age (years)		0 (0, 0)	0.678	0 (−0.1, 0)	0.317
Sex	Female	0.7 (0.4, 1.0)	<0.001	−0.7 (−1.7, 0.4)	0.221
Diagnosis	Reference: other headache				
	Migraine	0.5 (0.1, 0.9)	0.008	1.5 (0.1, 2.9)	0.035
	TTHA	0 (−0.4, 0.4)	0.854	1 (−0.4, 2.3)	0.153
Average frequency of headache attacks (times per month)	Reference: <1				
	1–3	−1.2 (−2.5, 0)	0.045	1.7 (−2.6, 6)	0.436
	4–11	−1.4 (−2.6, −0.2)	0.021	3.7 (−0.5, 7.9)	0.086
	≥12	−1.5 (−2.8, −0.3)	0.014	8.4 (4.2, 12.7)	<0.001
Average headache attack duration	Reference: <30 min				
	30 min to 4 h	0.2 (−0.3, 0.7)	0.384	0.7 (−1, 2.5)	0.393
	4–72 h	0.6 (0.1, 1.1)	0.011	1.2 (−0.4, 2.8)	0.151
	≥72 h	0.8 (0.3, 1.3)	0.001	4.3 (2.6, 5.9)	<0.001
Duration of headache history (days)		0 (0, 0)	0.27	0 (0, 0)	0.079
Frequency of painkillers /Peak pain intensity *		0 (0, 0.1)	<0.001	0.5 (0.3, 0.8)	<0.001

CI, confidence interval; coef, coefficient; TTHA, tension-type headache. * If the dependent variable is peak pain intensity, the coefficient and *p*-value refer to the effect of painkiller use, and vice versa.

## Data Availability

The datasets presented in this article are not readily available because the study is ongoing and data collection is still in progress. Requests to access the datasets should be directed to the corresponding author’s email address.

## Abbreviations

The following abbreviations are used in this manuscript:

AI	Artificial intelligence
HIT-6	Headache Impact Test-6
PHQ-9	Patient Health Questionnaire-9
GAD-7	Generalized Anxiety Disorder 7-item scale
t-SNE	t-distributed stochastic neighbor embedding
HDBSCAN	Hierarchical density-based spatial clustering of applications with noise
OLS	Ordinary least squares
TTHA	Tension-type headache

## Appendix A

**Table A1:** Raw HIT-6, PHQ-9, and GAD-7 scores used for clustering in 10 sample patients. HIT-6, Headache Impact Test-6; PHQ-9, Patient Health Questionnaire-9; GAD-7, Generalized Anxiety Disorder 7-Item Scale.

No	0	1	2	3	4	5	6	7	8	9
HIT-6	63	57	54	62	66	62	36	49	53	67
PHQ-9	4	9	5	5	4	17	4	4	7	16
GAD-7	4	7	6	3	4	1	0	1	2	8
